# Dexmedetomidine Leads to Less Emergence Delirium Compared to Midazolam in Pediatric Tonsillectomy and/or Adenoidectomy: A Systematic Review and Meta-Analysis

**DOI:** 10.7759/cureus.81686

**Published:** 2025-04-04

**Authors:** Pantea Jeizan, Kimia Baharimehr, Radin Kamvar, Asal Abolghasemi Fard, Seyedehhasti Zojaji, Amir Karimi, Ramin Zojaji

**Affiliations:** 1 Internal Medicine, Edward Via College of Osteopathic Medicine, Virginia, USA; 2 Medicine, University of California, Riverside, Riverside, USA; 3 Hospital Medicine, Albert Szent Gyorgy Health Center, University of Szeged, Szeged, HUN; 4 Department of Cellular and Molecular Biology, Faculty of Modern Science and Technologies, Tehran Medical Science, Islamic Azad University, Tehran, IRN; 5 Biomedical Sciences, Toronto Metropolitan University, Toronto, CAN; 6 Orthopedic Surgery, Case Western Reserve University School of Medicine, Cleveland, USA; 7 Otorhinolaryngology, Azad University, Tehran, IRN

**Keywords:** emergence delirium, midazolam, pediatric anesthesiology, post-tonsillectomy bleeding, dexmedetomidine

## Abstract

Tonsillectomy, with or without adenoidectomy, is a common pediatric surgical procedure that often induces significant preoperative anxiety, affecting both children and their parents. This anxiety can lead to aggressive behaviors, increased distress, and complicated postoperative pain management. Midazolam, often used for its rapid sedative effects, has drawbacks such as the potential for paradoxical reactions and respiratory depression. Alternatively, dexmedetomidine, known for its sedative, anxiolytic, and analgesic properties without significant respiratory depression, is becoming a favored option. The objective of this study is to compare the effectiveness of dexmedetomidine versus midazolam in reducing anxiety, emergence delirium (ED), and postoperative pain in pediatric patients undergoing these procedures. A comprehensive search was conducted using PubMed, MEDLINE, EBSCOhost, and Google Scholar to identify studies from January 1, 2000, to March 1, 2025, comparing the effects of dexmedetomidine and midazolam in pediatric patients undergoing tonsillectomy and/or adenoidectomy. Eligible studies were selected following PRISMA guidelines, with a focus on outcomes related to sedation levels, ED, pediatric anesthesia emergence delirium (PAED) score, use of analgesics, and duration of stay in the post-anesthesia care unit (PACU). Data were analyzed using a random-effects model to accommodate inter-study variability. The meta-analysis included six studies with a total of 668 participants. Dexmedetomidine was associated with significantly lower rates of ED, with an odds ratio (OR) of 0.43 (95% confidence interval (CI): 0.27-0.68, P < 0.01), and lower PAED scores compared to midazolam. Dexmedetomidine also demonstrated superior pain control, requiring less additional analgesia with an OR of 2.11 (95% CI: 1.42-3.12, P < 0.01). However, no significant differences were noted in anesthesia duration, extubation times, or PACU stays. Dexmedetomidine appears to be more effective than midazolam in reducing the incidence of ED and managing postoperative pain in children undergoing tonsillectomy and/or adenoidectomy, without extending recovery times. These findings support the preferential use of dexmedetomidine for pediatric premedication in Tonsillectomy and/or adenoidectomy, potentially improving patient outcomes and satisfaction while maintaining cost-effectiveness in surgical settings.

## Introduction and background

Tonsillectomy, with or without adenoidectomy, ranks among the most frequent surgical interventions in pediatric care [1]. This preoperative period often triggers significant anxiety in both children and their parents, posing a challenge for pediatric anesthesiologists to manage both pre- and postoperative anxiety effectively [2]. Anxiety not only can lead to aggressive behaviors and heightened distress but also complicates the management of postoperative pain [3]. Midazolam is commonly used as preanesthesia medication for children due to its rapid sedative effects and short duration of action [2,4]. However, it is not without drawbacks, including potential side effects like restlessness, paradoxical reactions, cognitive impairment, amnesia, and respiratory depression [5].

On the other hand, dexmedetomidine, a highly selective α-2 adrenoceptor agonist, has been gaining attention for its capacity to provide sedation, anxiolysis, and analgesia without the risk of significant respiratory depression, making it particularly attractive for pediatric premedication [6]. It allows for varying levels of sedation - from mild to deep - without deep sedation risks, and patients under its effect can easily be awakened yet return to a sleep-like state when undisturbed, mimicking natural sleep.

Emergence delirium (ED) is a notable complication of post-general anesthesia in children, with incidence rates ranging from 10% to 50% [7,8]. ED manifests as inconsolable crying, thrashing, disorientation, hallucinations, and cognitive impairment, often resolving spontaneously but potentially leading to self-injury, surgical site damage, increased nursing care costs, and caregiver dissatisfaction [9]. The effectiveness of midazolam versus dexmedetomidine in preventing ED remains debated. For example, Taneja et al. found that dexmedetomidine reduced ED in pediatric dental procedures compared to midazolam, whereas Cho et al. reported no difference between the two medications [10,11]. This discrepancy suggests that surgical or procedural specifics may influence the comparative outcomes of these drugs.

Given the prevalence of tonsillectomies as a common pediatric procedure, our study aims to directly compare the effectiveness of dexmedetomidine versus midazolam in managing pediatric patients undergoing tonsillectomy with or without adenoidectomy.

## Review

Methods

Search Strategy

The PubMed, MEDLINE, EBSCOhost, and Google Scholar electronic databases were searched on March 1, 2025, to identify all studies published between January 1, 2000, and March 1, 2025, that compared the effects of dexmedetomidine to midazolam in children undergoing tonsillectomy and/or adenoidectomy. The following keywords and Medical Subject Headings (MeSH) terms were used in combination with the “AND” or “OR” Boolean operators: (midazolam) AND (“dexmedetomidine” OR “Dex”) AND (“children” OR “child” OR “infant” OR “pediatrics” OR “adolescent”) AND (“tonsillectomy” OR “adenoidectomy” OR “Adenotonsillectomy”) AND (“randomized controlled trial” OR “RCT”).

Eligibility Criteria

We included articles that met the following criteria: 1) the availability of full-text manuscripts in English and 2) studies comparing the effects of dexmedetomidine with midazolam in children undergoing tonsillectomy and/or adenoidectomy. We excluded from our analysis 1) case reports, 2) systematic reviews, 3) studies duplicated across databases, 4) gray literature, including abstracts and preprint articles, and 5) publications in languages other than English.

Study Selection

**Figure 1:**
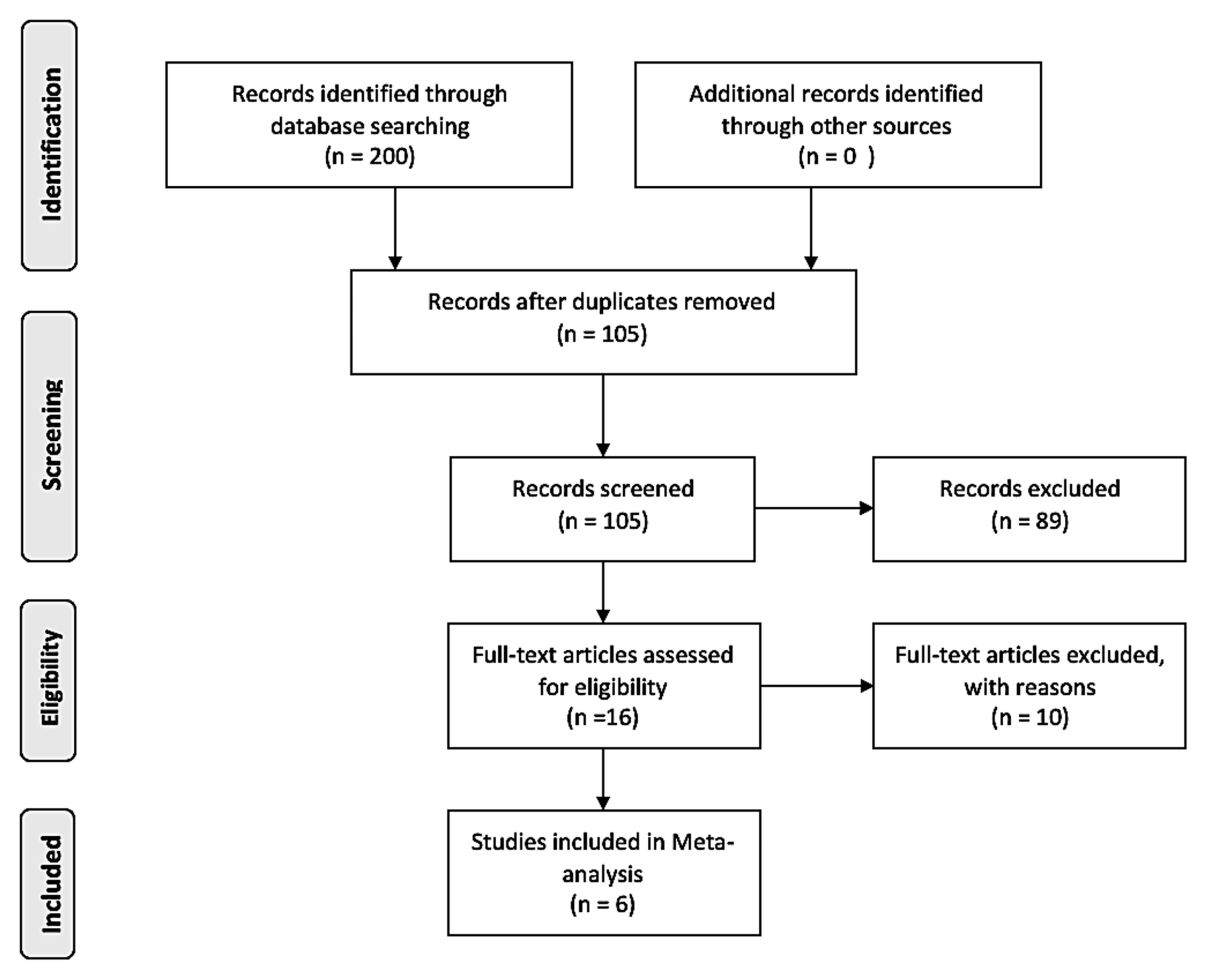
Preferred Reporting Items for Systematic Reviews and Meta-Analyses (PRISMA) diagram

This systematic review was meticulously conducted in accordance with the Preferred Reporting Items for Systematic Reviews and Meta-Analyses (PRISMA) guidelines [12]. Two reviewers (RK and AAF) independently assessed each article's eligibility, ensuring a rigorous evaluation process. In cases of disagreement, a third reviewer (AHK) was consulted to achieve consensus, thus maintaining the integrity of the selection process. Initially, our search identified 105 articles. After the removal of duplicates, these articles were subjected to a preliminary screening based on their titles and abstracts, aligning with our review's objectives. This screening process narrowed the field to 16 potentially relevant studies that warranted full-text examination. Of these, only six studies fulfilled our strict inclusion criteria and were selected for detailed quantitative analysis [11,13-17]. To ensure comprehensiveness, we also scrutinized the references within each selected article; however, this did not yield any additional studies for inclusion (Figure 1). It is important to note that studies excluded at the full-text stage were generally omitted because they either lacked full-text access, did not focus on the outcomes relevant to our study, or did not compare dexmedetomidine directly with midazolam, among other reasons. These criteria were stringently applied to focus our analysis on the most pertinent and high-quality data available.

Data Extraction

Data extraction for all studies that met the inclusion criteria was meticulously conducted by two independent reviewers (RK and SZ). Each study was thoroughly evaluated, and the data collected included a range of variables necessary for a comprehensive analysis. These variables comprised the author(s), year of publication, study design, data source, number of patients involved, and patient demographics such as mean age, weight, and height. In addition, detailed information regarding the method of sedative administration and the type of anesthetic used was gathered.

The primary outcomes evaluated in our systematic review were meticulously chosen to assess the efficacy and safety of sedative use in pediatric anesthesia. These outcomes included sedation levels in children, ED, scores on the Pediatric Anesthesia Emergence Delirium (PAED) scale, and the use of additional analgesics. We also critically examined the duration of stay in the post-anesthesia care unit (PACU), which is indicative of the recovery process, as well as the overall duration of anesthesia and extubation time.

It is important to note that not all studies provided data on each of these outcomes. Therefore, data extraction was carefully tailored to accommodate the availability of data within the included studies. This approach ensured that our analysis was both accurate and reflective of the current evidence base regarding the use of sedatives in pediatric patients undergoing anesthesia.

Quality Assessment

The quality of each study included in our meta-analysis was rigorously evaluated using the Cochrane Collaboration's risk of bias assessment tools, tailored for RCTs. This evaluation framework includes seven critical criteria: random sequence generation, allocation concealment, blinding of participants and personnel, blinding of outcome assessment, and completeness of outcome data, among others. Each study was assessed on these parameters, with potential biases categorized as “low,” “unclear,” or “high” risk [18]. This process was conducted independently by two reviewers (AAF and PJ), ensuring an unbiased approach. In instances of discrepancies between the reviewers, a third reviewer (AHK) was consulted to resolve the differences and achieve a consensus on the bias assessment.

Statistical Analysis

For our meta-analysis, we conducted computational analyses through the online platform MetaAnalysisOnline.com [19]. This platform allowed us to utilize advanced statistical tools tailored for meta-analytic research. Specifically, we employed a random-effects model to derive combined risk estimates, including ORs and their 95% CIs. This model was chosen because it accommodates the potential variability across studies, thus enhancing the robustness and generalizability of our results.

For continuous variables such as anesthesia time, we performed our analysis using the random-effects model coupled with the inverse variance method. This approach enabled us to compare the standardized mean differences (SMDs) across studies, providing a comprehensive overview of the effects measured. The random-effects model was particularly crucial here as it assumes that the studies included in the meta-analysis are sampled from populations that vary systematically, which is often the case in clinical research.

We also generated forest plots to visually represent both individual and aggregated study effects. These plots are instrumental in illustrating the size and direction of effects, aiding in the interpretation of how data distributions vary across studies and highlighting potential differences. This visual representation helps in assessing the consistency of study findings and in identifying any outliers.

Statistical significance was set at P < 0.05. To assess the heterogeneity among the included studies, which refers to the variation in study outcomes between the studies, we used I² and chi-square tests. We categorized heterogeneity as follows: not important (0-40%), moderate (40-60%), substantial (50-90%), and considerable (75-100%). This categorization helps in determining the reliability of the meta-analysis results and whether the observed effects could be attributed to underlying differences among the studies rather than chance.

Results

This study included six articles with 668 participants, of whom 347 were in the dexmedetomidine group and 321 in the midazolam group [11,13-17]. The characteristics of the studies included are provided in Table 1. The age of the study participants ranged from 0 to 12 years. The routes of administration of the sedative agents were either oral or intranasal. Of the six studies, two studies were performed in Egypt and one study in each of Turkey, South Korea, Oman, and China. The majority of the studies used sevoflurane and O_2_ as an anesthetic agent.

**Table 1:** Characteristics of the included articles Group P: placebo group; Group M: midazolam group; Group D: dexmedetomidine group; N/A: not applicable

Title	Author	Country	Year	Type of operation	ASA status	Age	Weight (kg)	Height (cm)	Groups	Timing of medication	Number of patients	Anesthesia
Dexmedetomidine vs midazolam for premedication of pediatric patients undergoing anesthesia	Akin et al. [13]	Turkey	2012	Adenotonsillectomy	I	2-9 years Group M: 6 (2–9) Group D: 5 (3–9)	Group M: 19.5 (11–35) Group D: 18.5 (11–35)	N/A	Group M: received 0.2 mg·kg−1 of intranasal midazolam. Group D: received 1 μg·kg−1 of intranasal dexmedetomidine.	45–60 min before the induction of anesthesia	90 patients, 45 in each group	Sevoflurane, NO, and O_2_
A placebo-controlled randomized trial comparing oral midazolam, dexmedetomidine, and gabapentin on prophylaxis of emergence agitation after sevoflurane anesthesia in adenotonsillectomy	Algyar et al. [14]	Egypt	2025	Adenotonsillectomy	I or II	3-10 years Group P: 6.3 ± 2.57 Group M: 7.1 ± 2.05 Group D: 6.4 ± 2.08 Group G: 6.1 ± 1.83	Group P: 26.8 ± 8.79 Group M: 28.9 ± 8.13 Group D: 26.6 ± 7.45 Group G: 25.7 ± 7.04	Group P: 117.9 ± 16.58. Group M: 122.5 ± 13.39. Group D: 118.2 ± 13.58. Group G: 116.5 ± 11.74	Group P: the placebo group. Group M: 0.5 mg/kg of dissolved midazolam. Group D: 4 µg/kg of dissolved DEX. Group 4: 10 mg/kg of dissolved gabapentin.	30 minutes prior to induction	240 patients, 60 in each group	Sevoflurane and O2
Comparison of single minimum dose administration of dexmedetomidine and midazolam for prevention of emergence delirium in children: a randomized controlled trial	Cho et al. [11]	South Korea	2019	Tonsillectomy	I or II	2-12 years Group M: 7.2 ± 2.2 years Group D: 6.7 ± 2.4 years	Group M: 28.9 ± 11.3 Group D: 26.3 ± 10.0	Group M: 124.2 ± 13.2. Group D: 120.6 ± 13.8	Group M: midazolam (0.03 mg/kg IV). Group D: dexmedetomidine (0.3 μg/kg IV)	Five minutes before the end of surgery	66 patients, 32 in midazolam group, 34 in DEX group	Thiopental sodium, meperidine, rocuronium, and sevoflurane
Preanesthetic medication in children: A comparison of intranasal dexmedetomidine versus oral midazolam	Ghali et al. [15]	Oman	2011	Adenotonsillectomy	I	4 to 12 years Group M : 8.1 ± 2.3 years Group D: 8.2 ± 1.4 Years	Group M: 17.9 ± 5.89 Group D: 18.4 ± 4.74	N/A	Group M received 0.5 mg/kg oral midazolam, Group D received 1 μg/kg intranasal dexmedetomidine	60 minutes before induction of anesthesia	120 patients, 60 in each group	Sevoflurane and O2
Intranasal dexmedetomidine versus intranasal midazolam as pre-anesthetic medication in pediatric age group undergoing adenotonsillectomy	Saad et al. [16]	Egypt	2020	Adenotonsillectomy	I	3−7 years Group M: 5.13 ± 1.54 years Group D: 5.04 ± 1.49 Years	Group M: 18.08 ± 3.09, Group D: 17.83 ± 2.78	N/A	Group M received intranasal midazolam (0.2 mg/kg), Group D received intranasal dexmedetomidine (1 μg/kg)	45 minutes before induction	48 patients, 24 in each group	Sevoflurane and O2
Effect of intranasal dexmedetomidine or midazolam for premedication on the occurrence of respiratory adverse events in children undergoing tonsillectomy and adenoidectomy: a randomized clinical trial	Shen et al. [17]	China	2022	Tonsillectomy and/or adenoidectomy	I or II	0 to 12 years	N/A	N/A	Group P: the placebo group. Group M: intranasal midazolam (0.1 mg/kg). Group D: intranasal DEX (2.0 μg/kg)	Upon arrival in the anesthetic preparation room, children received intranasal premedication at approximately 30 to 60 minutes.	373 patients, 125 patients in the placebo group, 124 patients in both the midazolam group and the DEX group	N/A

Quality Assessment

**Figure 2:**
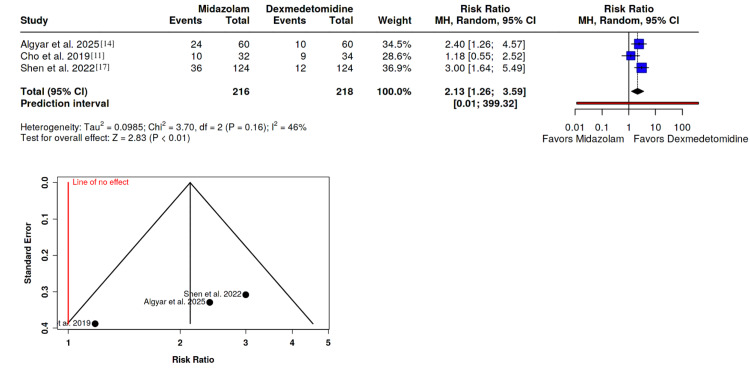
Forest plot depicting the impact of dexmedetomidine and midazolam on emergence delirium

The bias risk for the six RCTs was evaluated using the Cochrane Collaboration's risk-of-bias assessment tool. Random sequence generation showed a low risk of bias across all studies. Allocation concealment was assessed in five studies (83.3%), participant blinding in three (50%), and outcome assessment blinding in five (83.3%). In addition, incomplete outcome data and selective outcome reporting were noted in five studies (83.3%), and other sources of bias were identified in five studies (83.3%) (Figure 2).

Proportion of ED

The incidence of ED was analyzed using data from three studies, comprising 216 patients in the midazolam group and 218 in the dexmedetomidine group [11,14,17]. Patients receiving dexmedetomidine showed significantly lower odds of experiencing ED (OD: 2.13; 95% CI: 1.26 to 3.59; P < 0.01). Moderate heterogeneity was noted (I^2^ = 47%), and Egger's test indicated potential publication bias (P = 0.018), suggesting a need for cautious interpretation of the results (Figure 2).

PAED Score

**Figure 3:**
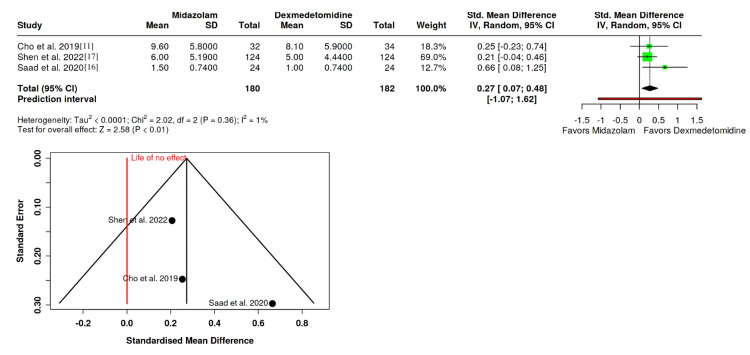
Forest plot depicting impact of dexmedetomidine and midazolam on pediatric anesthesia emergence delirium score

The PAED score was gathered from three RCTs involving 180 patients in the midazolam group and 182 in the dexmedetomidine group [11,16,17]. Results showed a significantly lower mean PAED score in the dexmedetomidine group (SMD: 0.27; 95% CI: 0.07 to 0.48; P < 0.01), with minimal heterogeneity among the studies (I^2^ = 1%). No significant funnel plot asymmetry was observed (P = 0.407), indicating robustness in the reported findings (Figure 3).

Pain Control

**Figure 4:**
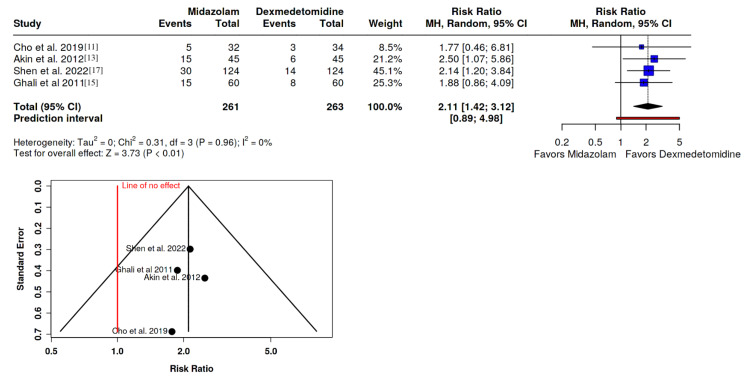
Forest plot depicting the impact of dexmedetomidine and midazolam on analgesic use

Pain control was evaluated by comparing the number of analgesic medications administered to patients across four RCTs, which included 261 patients in the midazolam group and 263 in the dexmedetomidine group [11,13,15,17]. The analysis showed that patients in the midazolam group required more analgesia, suggesting better pain control in the dexmedetomidine group (OD: 2.11; 95% CI: 1.42 to 3.12; P < 0.01). The studies exhibited uniformity in results, with no detected heterogeneity (I^2^ = 0%), and no significant funnel plot asymmetry was found (P = 0.693) (Figure 4).

Modified Observer’s Assessment of Alertness/Sedation

**Figure 5:**
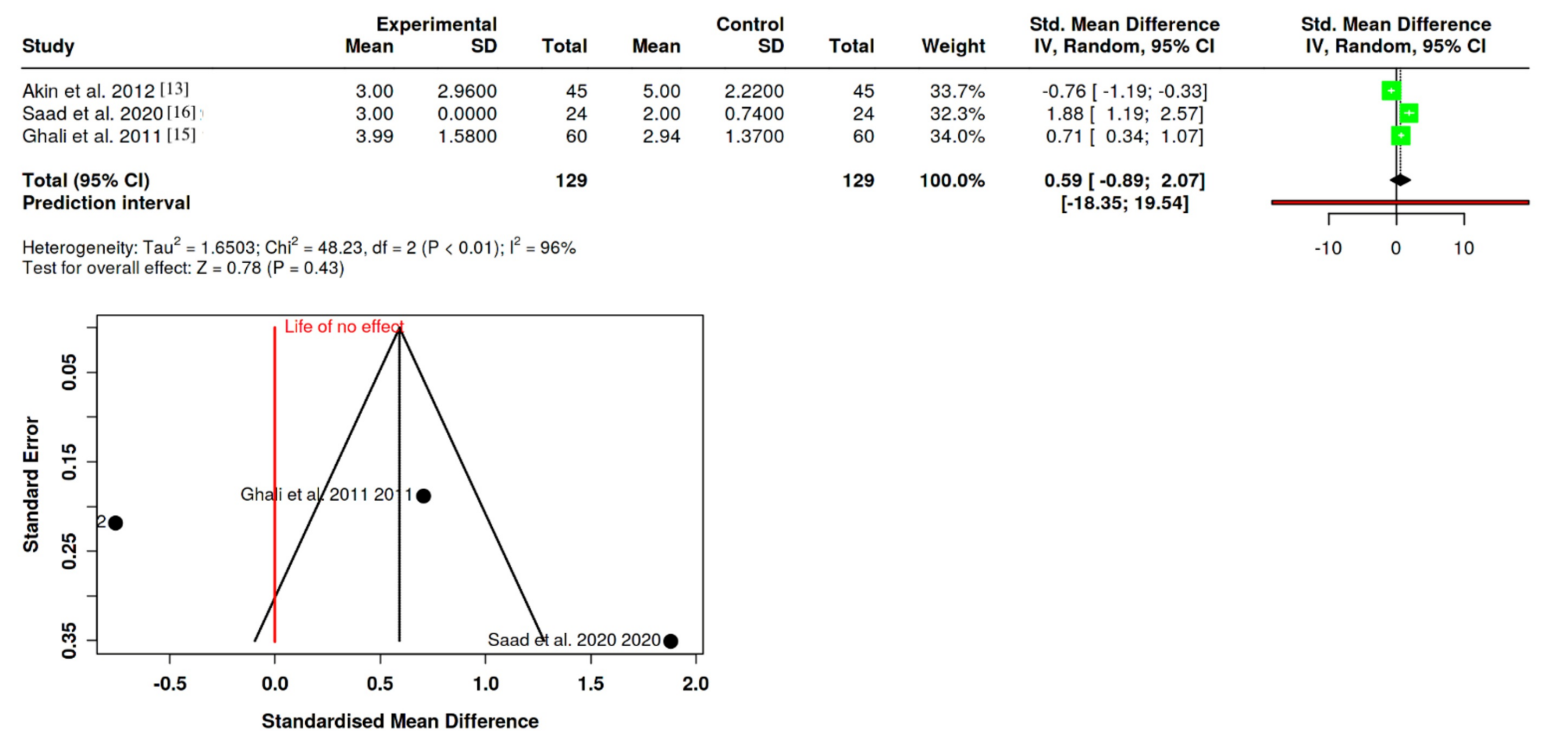
Forest plot depicting the impact of dexmedetomidine and midazolam on modified observer’s assessment of alertness/sedation

This assessment involved three RCTs with 129 patients per group [13,15,16]. The analysis indicated no statistical difference in alertness/sedation levels between the groups (SMD: 0.59; 95% CI: -0.89 to 2.07; P = 0.43). Notably, there was significant heterogeneity among the studies (I^2^ = 96%), suggesting substantial variation in observer assessments or patient reactions. The Egger's test does not support the presence of funnel plot asymmetry (P = 0.686) (Figure 5).

Anesthesia Time

**Figure 6:**
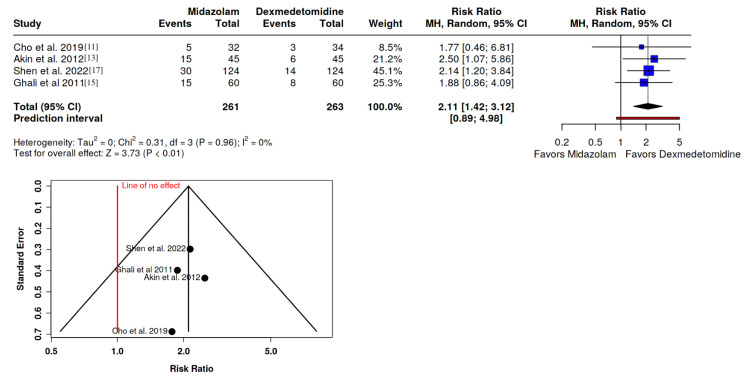
Forest plot depicting the impact of dexmedetomidine and midazolam on anesthesia time

The duration of anesthesia was analyzed using data from four RCTs, which included 261 patients in the midazolam group and 263 in the dexmedetomidine group [11,13,14,17]. Utilizing a random-effects model with an Inverse variance method, the SMD showed no statistical difference between the two groups (SMD: 0.07; 95% CI: -0.17 to 0.3; P = 0.44), indicating that neither drug significantly affects anesthesia duration. In addition, there was no evidence of heterogeneity among the studies (I^2^ = 0%), and Egger's test indicated no significant funnel plot asymmetry (P = 0.24), suggesting a balanced representation across the studies (Figure 6). 

Extubation Time

**Figure 7:**
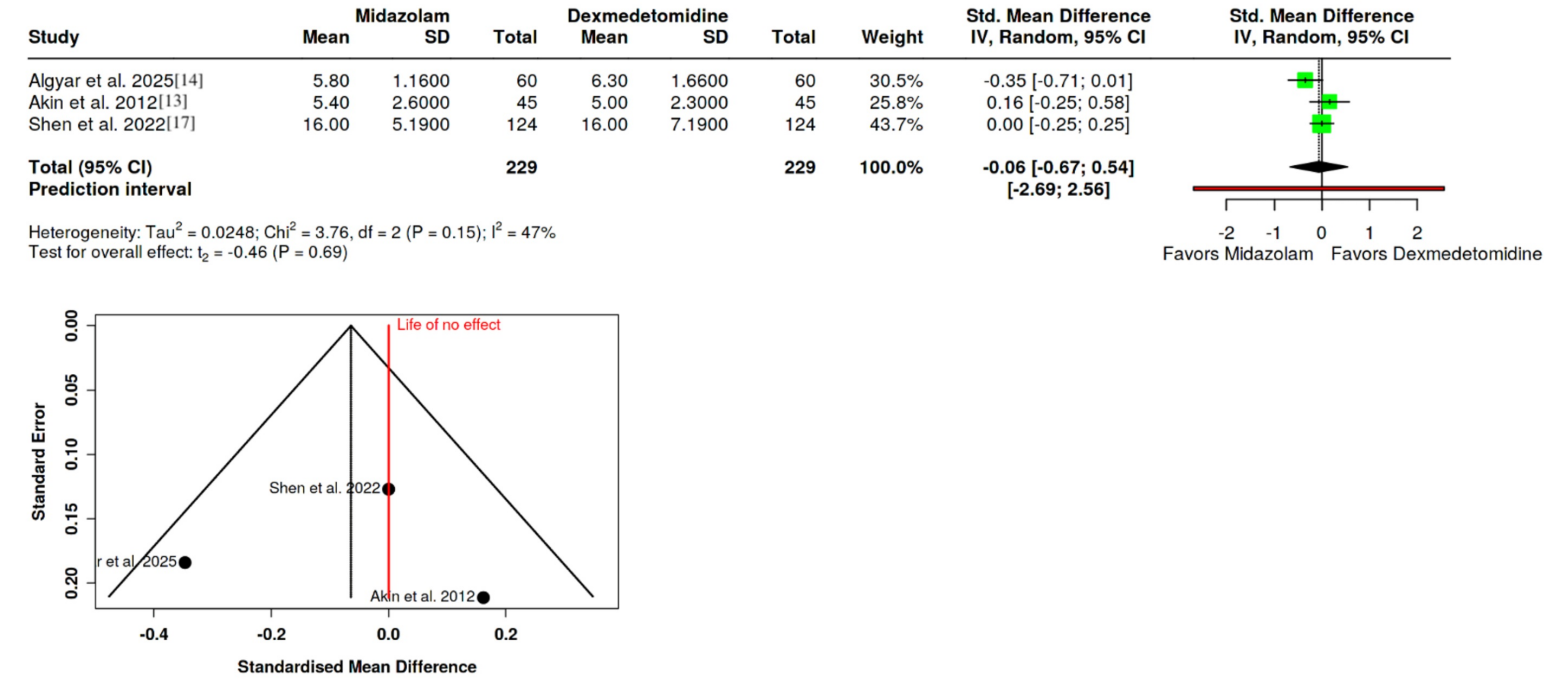
Forest plot depicting the impact of dexmedetomidine and midazolam on extubation time

The analysis of extubation time included data from three RCTs, each encompassing 229 patients in both the midazolam and dexmedetomidine groups [13,14,17]. The comparison using a random-effects model revealed no significant difference in extubation times (SMD: -0.06; 95% CI: -0.67 to 0.54; P = 0.69). However, moderate heterogeneity was detected among these studies (I^2^ = 47%), which might suggest varying extubation protocols or patient responses across studies. Egger's test did not indicate funnel plot asymmetry (P = 0.938), affirming the analytical balance (Figure 7).

PACU Stay

**Figure 8:**
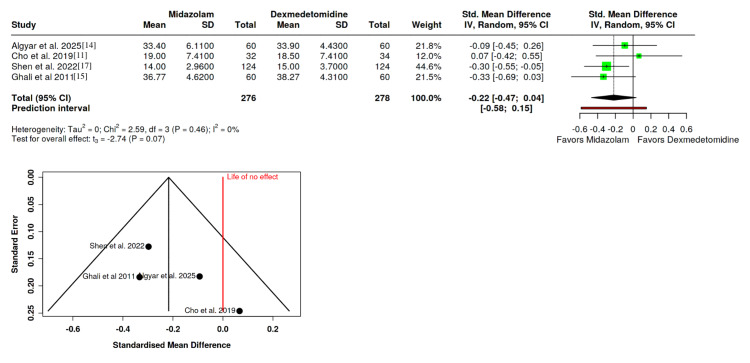
Forest plot depicting the impact of dexmedetomidine and midazolam on post-anesthesia care unit (PACU) time

The PACU stay duration was assessed in four RCTs with 276 patients in the midazolam group and 278 in the dexmedetomidine group [11,14,15,17]. The results, analyzed via a random-effects model, showed no significant difference (SMD: -0.22; 95% CI: -0.47 to 0.04; P = 0.07). Although not statistically significant, there was a trend suggesting a slightly shorter PACU stay in the midazolam group. No heterogeneity was observed among the participating studies (I^2^ = 0%), and Egger's test confirmed no evidence of funnel plot asymmetry (P = 0.241) (Figure 8). 

 *Quality Assessment*

**Figure 9:**
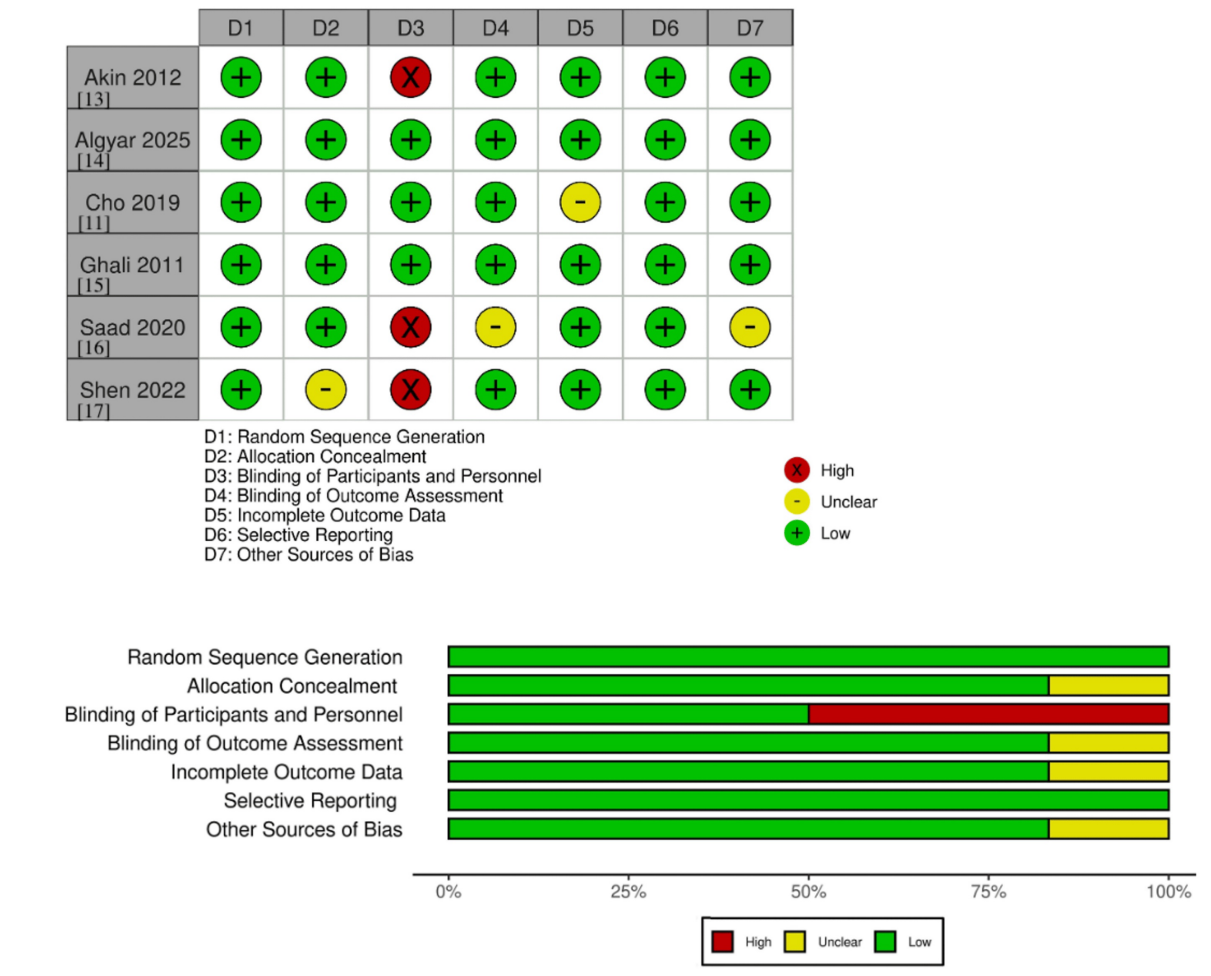
Summary risk assessment of the literature bias

The bias risk for the six RCTs was evaluated using the Cochrane Collaboration's risk-of-bias assessment tool. Random sequence generation showed a low risk of bias across all studies. Allocation concealment was assessed in five studies (83.3%), participant blinding in three (50%), and outcome assessment blinding in five (83.3%). In addition, incomplete outcome data and selective outcome reporting were noted in five studies (83.3%), and other sources of bias were identified in five studies (83.3%) (Figure 9).

Discussion

Children experiencing extreme anxiety and fear during anesthesia induction can face several adverse clinical outcomes, such as post-awakening delirium, a heightened need for analgesics, and various postoperative adverse reactions. Midazolam, renowned for its safety and easy oral administration, has traditionally been the preferred premedication for pediatric patients. However, ongoing research has explored various drugs and administration methods to identify an optimal premedication. Based on our study, both dexmedetomidine and midazolam provide similar effectiveness in terms of anesthesia duration, extubation times, and durations of stay in the PACU for pediatric patients undergoing tonsillectomy and/or adenoidectomy. Nonetheless, dexmedetomidine shows superior results in pain management, with the midazolam group having an OR of 2.11 for requiring additional analgesia compared to the dexmedetomidine group. Furthermore, dexmedetomidine significantly reduces the likelihood of ED and achieves lower PAED scores than midazolam, indicating a more effective control of anesthesia-related complications.

The latest inhalational anesthetic agents, such as desflurane and sevoflurane, are favored for their rapid recovery and washout characteristics. However, using these drugs as sole anesthetic agents is linked with an approximate 80% increase in the incidence of ED, which can lead to potential injuries among patients and medical staff, along with other postoperative complications [20]. This underscores the critical importance of effectively preventing ED, particularly in pediatric patients. The role of midazolam in preventing ED remains debated, with some studies identifying it as a risk factor for ED, while others support its use as a preventive strategy [21,22]. In contrast, our study found that the dexmedetomidine group exhibited not only lower rates of ED but also significantly reduced PAED scores compared to the midazolam group. These findings mirror those in adult populations, such as in a study by Riker et al., which compared the effects of dexmedetomidine and midazolam in 375 ICU patients on mechanical ventilation. The study reported lower delirium prevalence with dexmedetomidine treatment-54% compared to 76.6% with midazolam (P < .001) [23]. Similar trends have been observed in pediatric patients undergoing dental procedures, suggesting that dexmedetomidine might be a more effective option than midazolam for preventing ED [24].

The etiology of ED is complex and influenced by multiple factors. Understanding and modifying these causative factors, where possible, is crucial in reducing the incidence of ED and preventing its adverse consequences. One significant factor linked to ED is the severity of postoperative pain. Research by Kim et al. demonstrated that patients experiencing postoperative pain rated greater than 5 on the numerical rating scale were significantly more likely to develop ED [25]. Furthermore, a systematic review and meta-analysis by He et al. highlighted that patients with postoperative pain had an OR of 2.72 for developing ED [26]. Our study’s findings of reduced rates of ED and lower PAED scores in the dexmedetomidine cohort might be attributed to its effectiveness in minimizing postoperative pain. This is supported by our data showing that patients receiving midazolam had an OR of 2.11 for requiring additional analgesia compared to those treated with dexmedetomidine. This aligns with other studies indicating that dexmedetomidine not only provides sufficient analgesia but also decreases the need for additional pain medications [27]. Thus, dexmedetomidine could be preferable in settings where minimizing postoperative pain and reducing the risk of ED are critical objectives.

Keles et al. found that after dental procedures performed under general anesthesia, the duration of anesthesia and sedation scores were similar between patients who received dexmedetomidine and those who received midazolam [24]. Similarly, Peng et al. observed no significant differences in recovery time or the length of stay in the PACU between the two groups [6]. These findings align with our own study, which also showed no differences in anesthesia duration, extubation times, and PACU stays for pediatric patients undergoing tonsillectomy and/or adenoidectomy. While the specific financial impact of ED remains unquantified, operating rooms are estimated to cost approximately $36-37 per minute, and recovery rooms are about $9 per minute [28]. In addition, the costs associated with treating injuries, increased staff demands, and the potential need for additional sedative medications can significantly add to the economic burden of ED [29]. Considering these factors, dexmedetomidine may offer a more cost-effective and clinically efficient option for premedication before tonsillectomy and/or adenoidectomy than midazolam.

This study is not without some limitations that warrant consideration. One significant limitation concerns the variability in medication timing across the studies included, which could potentially influence the effectiveness of the medications on the measured outcomes. Differences in timing may affect the onset of action and interactions with other anesthetic agents, possibly skewing results. Another limitation is the reliance on a limited number of studies, which might not fully represent the range of clinical settings and patient populations. This could affect the robustness and generalizability of the findings. In addition, the meta-analysis focused predominantly on quantitative data, potentially overlooking the qualitative aspects of patient experiences that could provide deeper insights into the subjective effects of the medications. The study also did not account for the potential influence of inter-study variations in anesthetic techniques and patient care standards, which might have contributed to the observed differences in outcomes. Despite these limitations, by combining data from all included RCTs, we analyzed a large population of patients, enhancing the generalizability of the results across different settings and populations. The significant differences observed in key outcomes, such as the incidence of ED and PAED scores, provide valuable insights that could influence the choice of premedication in pediatric surgeries. These strengths affirm the importance and accuracy of the study's conclusions.

## Conclusions

Our study underscores the effectiveness of dexmedetomidine over midazolam in pediatric anesthesia, particularly for managing postoperative pain and reducing the incidence of ED. While both medications showed similar efficacy in terms of anesthesia duration and recovery times, dexmedetomidine significantly outperformed midazolam in minimizing the need for additional analgesia and lowering PAED scores. These findings suggest that dexmedetomidine could be a more advantageous premedication choice, offering better control of anesthesia-related complications and enhancing overall patient safety in pediatric surgeries such as tonsillectomy and/or adenoidectomy. The economic benefits, coupled with clinical outcomes, advocate for a reevaluation of premedication protocols to optimize care in pediatric anesthetic practice.

## References

[1] Deutsch E (1996). Tonsillectomy and adenoidectomy: changing indications. Pediatr Clin North Am.

[2] Kogan A, Katz J, Efrat R, Eidelman LA (2002). Premedication with midazolam in young children: a comparison of four routes of administration. Paediatr Anaesth.

[3] Egan KJ, Ready LB, Nessly M, Greer BE (1992). Self-administration of midazolam for postoperative anxiety: a double blinded study. Pain.

[4] Kain ZN, Hofstadter MB, Mayes LC, Krivutza DM, Alexander G, Wang SM, Reznick JS (2000). Midazolam: effects on amnesia and anxiety in children. Anesthesiology.

[5] Bergendahl H, Lönnqvist PA, Eksborg S (2005). Clonidine: an alternative to benzodiazepines for premedication in children. Curr Opin Anaesthesiol.

[6] Peng K, Wu SR, Ji FH, Li J (2014). Premedication with dexmedetomidine in pediatric patients: a systematic review and meta-analysis. Clinics (Sao Paulo).

[7] Dahmani S, Stany I, Brasher C (2010). Pharmacological prevention of sevoflurane- and desflurane-related emergence agitation in children: a meta-analysis of published studies. Br J Anaesth.

[8] Kuratani N, Oi Y (2008). Greater incidence of emergence agitation in children after sevoflurane anesthesia as compared with halothane: a meta-analysis of randomized controlled trials. Anesthesiology.

[9] Ali MA, Abdellatif AA (2013). Prevention of sevoflurane related emergence agitation in children undergoing adenotonsillectomy: a comparison of dexmedetomidine and propofol. Saudi J Anaesth.

[10] Taneja S, Jain A (2023). Systematic review and meta-analysis comparing the efficacy of dexmedetomidine to midazolam as premedication and a sedative agent in pediatric patients undergoing dental procedures. Oral Maxillofac Surg.

[11] Cho EA, Cha YB, Shim JG, Ahn JH, Lee SH, Ryu KH (2020). Comparison of single minimum dose administration of dexmedetomidine and midazolam for prevention of emergence delirium in children: a randomized controlled trial. J Anesth.

[12] Moher D, Liberati A, Tetzlaff J, Altman DG (2009). Preferred reporting items for systematic reviews and meta-analyses: the PRISMA statement. PLoS Med.

[13] Akin A, Bayram A, Esmaoglu A, Tosun Z, Aksu R, Altuntas R, Boyaci A (2012). Dexmedetomidine vs midazolam for premedication of pediatric patients undergoing anesthesia. Paediatr Anaesth.

[14] Algyar M, Abdelghany A, Arafa S, Elsayed A (2025). A placebo-controlled randomized trial comparing oral midazolam, dexmedetomidine, and gabapentin on prophylaxis of emergence agitation after sevoflurane anesthesia in adenotonsillectomy. Pain Physician.

[15] Ghali AM, Mahfouz AK, Al-Bahrani M (2011). Preanesthetic medication in children: a comparison of intranasal dexmedetomidine versus oral midazolam. Saudi J Anaesth.

[16] Saad B, Tharwat A, Ghobrial H, Elfawal S (2020). Intranasal dexmedetomidine versus intranasal midazolam as pre-anesthetic medication in pediatric age group undergoing adenotonsillectomy. Ain Shams J Anesthes.

[17] Shen F, Zhang Q, Xu Y (2022). Effect of intranasal dexmedetomidine or midazolam for premedication on the occurrence of respiratory adverse events in children undergoing tonsillectomy and adenoidectomy: a randomized clinical trial. JAMA Netw Open.

[18] (2025). Cochrane handbook for systematic reviews of interventions. https://training.cochrane.org/handbook/current.

[19] Fekete JT, Győrffy B (2025). MetaAnalysisOnline.com: web-based tool for the rapid meta-analysis of clinical and epidemiological studies. J Med Internet Res.

[20] Farag RS, Spicer AC, Iyer G, Stevens JP, King A, Bain PA, McAlvin JB (2024). Incidence of emergence agitation in children undergoing sevoflurane anesthesia compared to isoflurane anesthesia: an updated systematic review and meta-analysis. Paediatr Anaesth.

[21] Almenrader N, Galante D, Engelhardt T (2014). Emergence agitation: is there a European consensus?. Br J Anaesth.

[22] Urits I, Peck J, Giacomazzi S (2020). Emergence delirium in perioperative pediatric care: a review of current evidence and new directions. Adv Ther.

[23] Riker RR, Shehabi Y, Bokesch PM (2009). Dexmedetomidine vs midazolam for sedation of critically ill patients: a randomized trial. JAMA.

[24] Keles S, Kocaturk O (2018). Comparison of oral dexmedetomidine and midazolam for premedication and emergence delirium in children after dental procedures under general anesthesia: a retrospective study. Drug Des Devel Ther.

[25] Kim HJ, Kim DK, Kim HY, Kim JK, Choi SW (2015). Risk factors of emergence agitation in adults undergoing general anesthesia for nasal surgery. Clin Exp Otorhinolaryngol.

[26] He M, Zhu Z, Jiang M (2024). Risk factors for postanesthetic emergence delirium in adults: a systematic review and meta-analysis. J Neurosurg Anesthesiol.

[27] Prabhu MK, Mehandale SG (2017). Comparison of oral dexmedetomidine versus oral midazolam as premedication to prevent emergence agitation after sevoflurane anaesthesia in paediatric patients. Indian J Anaesth.

[28] Childers CP, Maggard-Gibbons M (2018). Understanding costs of care in the operating room. JAMA Surg.

[29] Huang J, Chopra N, Yepuri N, Kinthala S (2023). Emergence agitation and anesthetic considerations in the management of patients with post-traumatic stress disorder: a report of two cases and a review of the literature. Cureus.

